# *Stella* Regulates the Development of Female Germline Stem Cells by Modulating Chromatin Structure and DNA Methylation

**DOI:** 10.7150/ijbs.69240

**Published:** 2022-04-18

**Authors:** Changliang Hou, Xinyan Zhao, Geng G. Tian, Ji Wu

**Affiliations:** 1 Bio-X Institutes, Key Laboratory for the Genetics of Developmental and Neuropsychiatric Disorders, Ministry of Education, Shanghai Jiao Tong University, Shanghai 200240, China.; 2 Key Laboratory of Fertility Preservation and Maintenance of Ministry of Education, School of Basic Medical Sciences, Ningxia Medical University, Yinchuan 750004, China.; 3 Shanghai Key Laboratory of Reproductive Medicine, Shanghai 200025, China

**Keywords:** *Stella*, female germline stem cells, epigenomics, chromatin structure, DNA methylation

## Abstract

Female germline stem cells (FGSCs) have the ability to self-renew and differentiate into oocytes. *Stella*, encoded by a maternal effect gene, plays an important role in oogenesis and early embryonic development. However, its function in FGSCs remains unclear. In this study, we showed that CRISPR/Cas9-mediated knockout of *Stella* promoted FGSC proliferation and reduced the level of genome-wide DNA methylation of FGSCs. Conversely, *Stella* overexpression led to the opposite results, and enhanced FGSC differentiation. We also performed an integrative analysis of chromatin immunoprecipitation followed by high-throughput sequencing (ChIP-seq), high-throughput genome-wide chromosome conformation capture (Hi-C), and use of our published epigenetic data. Results indicated that the binding sites of STELLA and active histones H3K4me3 and H3K27ac were enriched near the TAD boundaries. Hi-C analysis showed that *Stella* overexpression attenuated the interaction within TADs, and interestingly enhanced the TAD boundary strength in STELLA-associated regions. Taking these findings together, our study not only reveals the role of *Stella* in regulating DNA methylation and chromatin structure, but also provides a better understanding of FGSC development.

## Introduction

In recent years, the incidence of infertility has gradually increased, becoming a global health problem 1. A drastic decline in the number of oocytes and poor oocyte quality are the main causes of female infertility. As a type of germline stem cell, female germline stem cells (FGSCs) possess the capacity for self-renewal and differentiation into oocytes 2-4. Moreover, our previous research showed that FGSCs were a type of germ cells between primordial germ cells (PGCs) and germinal vesicle (GV) oocytes 5, which were genetically close to PGCs, but had its unique molecular characteristics. Recent studies have revealed the mechanisms by which FGSC proliferation, differentiation, and apoptosis are regulated 2, 6-8. Ma et al. revealed that the PI3K-AKT pathway is important for FGSC maintenance 5. *Etv5*, *Foxo1*, and *Akt* were also shown to positively regulate FGSC self-renewal 9. In addition, Zou et al. evaluated different differentiation conditions of mouse FGSCs *in vitro*, and successfully differentiated FGSCs into GV oocytes 8. In mammals, genome-wide epigenetic reprogramming occurs in the early germline to erase parental epigenetic memories and promote germ cell differentiation 10, 11. Our previous study found that DNA methylation is involved in the unipotency of FGSCs and maintains their sexual identity 12. Zhao et al. showed that *Usp7* regulates the self-renewal and differentiation of FGSCs through DNA methylation 13. In addition, long noncoding RNA 14, 15, Circular RNA 16, and m6A modification 17 have been reported to participate in the epigenetic regulation of FGSCs development. These findings lay the foundation for further research on the mechanism of FGSC development.

*Stella* (also known as Dppa3 or PCG7) is a small protein composed of 150 amino acids, which is predominantly expressed in germ cells, early embryos, and pluripotent cells 18, 19. Accumulating evidence has indicated that *Stella* plays a significant role in mouse oocytes and early embryonic development. Liu et al. showed that *Stella* deficiency dramatically inhibits oocytes from the non-surrounded nucleolus (NSN) stage to the surrounded nucleolus (SN) stage 20. Embryos derived from *Stella*-deficient oocytes were also found to be arrested at the four-cell stage and fail to produce offspring 19, 21. In addition to its role in mouse oocytes and embryos, *Stella* also affects the differentiation of human embryonic stem cells 22 and tumor cells 23. Recent studies in our laboratory have shown that, after overexpressing *Stella*, *H19*, and *Zfp57,* and knockdown of *Plzf* in spermatogonial stem cells (SSCs), SSCs could be converted into induction of germline stem cells, which had similar morphology, DNA methylation pattern, and 3D chromatin structure as FGSCs 9. This finding suggests *Stella* is an important maternal-origin gene. However, to the best of our knowledge, no reports have been published about the possible role of *Stella* in FGSC development.

Several studies have shown that *Stella* protects DNA methylation status in the maternal pronucleus 24, 25. Bian and Yu demonstrated that a lack of *Stella* induces the loss of DNA methylation at imprinting loci 26. In addition, Singer et al. showed that *Stella* regulates pluripotency by maintaining the hypomethylated state of DNA in pluripotent stem cells 27. Additionally, *Stella* deficiency also impacts on heterochromatin-related factors HP1β. In *Stella*-null GV oocytes, pericentric heterochromatin HP1β was found to have lower staining intensity, and repressive histone marks H3K9me3 and H3K27me3 were more sparsely distributed 20. Moreover, chromocenter formation and major satellite RNA were impaired in *Stella*-deficient embryos 28. Both HP1β and major satellite RNA are reported to play roles in three-dimensional (3D) chromatin organization 29-31.

Recently, high-throughput genome-wide chromosome conformation capture (Hi-C) technology, which was developed from chromosome conformation capture (3C), has been used to visualize 3D chromatin organization at an unprecedentedly high resolution 32, 33. Hi-C studies have revealed that eukaryotic genomes are spatially organized into A/B-type compartments, topologically associated domains (TADs), and a chromatin loop 34, 35. The 3D chromatin structure is believed to regulate gene expression by establishing loops between enhancers and promoters 36. However, whether *Stella* plays a role in 3D chromatin organization remains unclear. In this study, we carried out an integrative analysis of chromatin immunoprecipitation followed by high-throughput sequencing (ChIP-seq), high-throughput genome-wide chromosome conformation capture (Hi-C), and the use of DNA methylation and RNA-seq data. Our results demonstrate that *Stella* regulates both DNA methylation and higher-order chromatin structure, accompanied by changes in gene expression.

## Materials and methods

### Culture of female germline stem cells

FGSCs were cultured in accordance with our previously published reports 2, 37. In brief, dissected muse ovarian tissues was digested by trypsin and collagenase Ⅳ respectively, and then further purified by magnetic beads coupled with MVH antibody. The purified FGSCs were cultured on mitomycin C-treated (10 μg/ml, Sigma) mitotically inactivated mouse STO cell feeders in 48-well plates at 37℃ with 5% CO_2_. They were passaged every 3-4 days at a ratio of 1:4 and the medium was replaced every 2 days.

### Generation of* Stella*-overexpressing FGSC lines

*Stella*-overexpressing FGSC lines were generated as previously described 9. Briefly, gene coding sequences were amplified and cloned into the multiple cloning site of lentivirus overexpression vectors. Lentivirus packaging was performed by OBiO Technology Co., Ltd. (Shanghai, China). When the FGSCs had grown to approximately 50% confluence, they were infected with lentivirus at an MOI of 60. At 24 h post-infection, the medium was replaced with fresh medium. Puromycin (1 μg/mL) was added to obtain stable FGSC lines. Empty lentiviral vector was used as a negative control.

### Generation of *Stella*-knockout FGSC lines

To generate CRISPR knockout cells, two different pairs of sgRNAs targeting *Stella* were designed 38. The sgRNAs were cloned into the pLenti-U6-spgRNA v2.0 vector. Lentivirus packaging and infection were conducted as described above. Subsequently, lentivirus-infected cells were isolated, serially diluted, and plated into 96-well plates. Single-cell clones were expanded and the genotype of each clone was verified using PCR and Sanger sequencing. The sequences of the sgRNAs used in this study are listed in Table S1.

### Off-target analysis

Off-target sites were predicted by an online design tool (http://crispr.mit.edu/). The PCR products containing off-target sites were confirmed by T7E1 enzyme digestion and Sanger sequencing 39. Primers for off-target sites are listed in Table S2.

### Cell viability assay

Cell viability assay was performed using the CCK-8 kit (Beyotime, Shanghai, China). In accordance with the manufacturer's protocol, FGSCs were seeded into 96-well plates at a density of 4 × 10^3^ cells per well. After incubation for 24 h, 20 µL of CCK-8 reagent was added into each well and incubated for 2 h. Optical density at 450 nm was measured on a microplate reader.

### Cell proliferation assay

Cell proliferation rate was analyzed using the EdU Apollo^®^567 *In vitro* Imaging Kit (RiboBio, Guangzhou, China). Cells were plated into 48-well plates at a density of 1 × 10^5^ cells per well. One day after seeding, the culture medium was exchanged for fresh medium containing 50 µM EdU solution and incubated for another 2h. Cells were fixed for 30 min in 4% paraformaldehyde (PFA) and permeabilized with 0.5% Triton X-100, and then neutralized with 2 mg/mL glycine for 5 min. After washing three times, cells were reacted with Apollo reaction mixture for 30 min, stained with Hoechst 33342 for 10 min, and visualized under a fluorescent microscope.

### Cell cycle analysis

Cell cycle analysis was conducted using the Cell Cycle and Apoptosis Analysis kit (Beyotime, Shanghai, China). Cells were washed twice with precooled phosphate-buffered saline (PBS), digested using trypsin, and fixed in 70% ethanol at 4°C overnight. Subsequently, the fixed cells were washed with precooled PBS and resuspended in PBS containing propidium iodide (PI) and RNase A at 37°C for 30 min. The cell cycle distribution was examined using a CytoFLEX flow cytometer (Beckman Coulter, Brea, CA, USA) and analyzed using FlowJo software.

### Reverse-transcription PCR (RT-PCR) and quantitative real-time PCR (qRT-PCR)

Total RNA was extracted with Trizol Reagent (Invitrogen, Carlsbard, CA, USA) and reverse-transcribed to cDNA. qRT-PCR was performed with ABI 7500 Real-Time System and the data were analyzed using 7500 Software. Primers for RT-PCR and qRT-PCR are listed in Table S3.

### Immunofluorescence staining

The double staining of MVH and STELLA was conducted as follows. FGSCs were fixed with 4% PFA for 30 min at room temperature. They were then incubated in blocking solution containing 10% normal goat serum for 60 min, and incubated with mouse monoclonal anti-MVH (1:100, Abcam, Cambridge, UK) overnight at 4℃. Cells were washed once with PBS and permeabilized with 0.5% Triton X-100 for 30 min. Next, the cells were incubated with rabbit polyclonal anti-STELLA (1:200, Abcam, Cambridge, UK) overnight at 4℃. After washing with PBS three times, the cells were incubated with the secondary antibody for 60 min, washed, and then incubated with DAPI for 10 min. Images were acquired using a Leica digital camera.

For OCT4 staining, before incubation in blocking buffer, cells were permeabilized with 0.5% Triton X-100 for 30 min and then washed with PBS three times. The remaining steps were the same as for the above double staining. The primary antibody was rabbit polyclonal anti-OCT4 (1:100, Santa Cruz, CA, USA).

### Western blot analysis

FGSCs were lysed in RIPA buffer containing protease inhibitor cocktail. Equal amounts of total proteins were denatured and separated on SDS-PAGE and transferred to PVDF membranes. Blocking of the membranes was performed in 5% non-fat powdered milk in Tris-buffered saline with Tween 20 (TBST) for 1 h. After blocking, the membranes were incubated with primary antibodies to STELLA (1:200, Abcam, Cambridge, UK), STRA8 (1:1000, Bioss, Beijing, China) overnight at 4°C. The membranes were washed with TBST three times and incubated with the secondary antibody at room temperature for 1 h. The labeled proteins were visualized using ECL reagents. The grayscale value was measured using ImageJ software.

### Dot blotting

Dot blots were conducted as described previously 40. Briefly, genomic DNA was extracted and subjected to denaturation. Then, equal concentrations of each sample were dotted on nitrocellulose blotting membrane and subjected to UV-crosslinking after air-drying. The membranes were blocked in 5% non-fat powdered milk in PBST for 60 min. Subsequently, the membranes were incubated with mouse anti-5mC (1:1000, Diagenode, Seraing, Belgium), followed by secondary antibody. The ECL kit was used to visualize the blots.

### RNA-sequencing (RNA-seq)

Total RNA was extracted from cells using Trizol Reagent (Invitrogen, Carlsbard, CA, USA). RNA quantity and quality were determined with 2100 Bioanalyzer (Agilent Technologies). RNA-Seq libraries was prepared using the KAPA Stranded mRNA-Seq kit (KAPA Biosystems, Wilmington, MA, USA), following the manufacturer's protocol. For bulk RNA-seq, approximately 2μg of total RNA (1×10^6^ cells) was used as starting material for each reaction.

### Chromatin immunoprecipitation followed by high-throughput sequencing (ChIP-seq)

ChIP-seq experiments were performed as described previously 41. Briefly, cells were cross-linked, lysed, and sheared to obtain 200-800 bp fragments. Chromatin was immunoprecipitated with antibodies against STELLA (2 μg, Abcam, Cambridge, UK) or rabbit IgG (2μg, Cell Signaling Technology, MA, USA). Library construction, purification, and next-generation sequencing were conducted as described above.

### Methylated DNA immunoprecipitation (MeDIP)

MeDIP experiments were performed using a MagMEDIP kit (Diagenode, Seraing, Belgium). Briefly, genomic DNA (gDNA) was extracted from FGSCs and sonicated to shear chromatin. Fifty nanograms of fragmented genomic DNA was used for the library construction and purified. Methylated DNA was immunoprecipitated with anti-5-methylcytosine antibody overnight at 4℃. After washing and elution, immunoprecipitated DNA was amplified and subjected to Illumina sequencing.

### *In situ* Hi-C

*In situ* Hi-C was performed as previously reported 9, 32. Briefly, cells were fixed, lysed, and digested with Dpn II restriction enzyme. Biotin was incorporated into the sticky ends of fragments before ligation. Proximity ligation was carried out with T4 DNA ligase. Then, DNA purification was carried out and the DNA was sheared into fragments. End repair, adenylation, and adapter ligation were performed using NEBNext End Repair Kit (NEB, Ipswich, MA, USA). Biotin-labeled fragments were pulled down using MyOne Streptavidin T1 beads (Life Technologies, Waltham, MA, USA). Hi-C DNA was amplified using the KAPA HiFi Library Amplification Kit (KAPA Biosystems, Wilmington, MA, USA). DNA size selection was performed using AMPure XP beads (Beckman Coulter, Brea, MA, USA). The concentration of the Hi-C libraries was determined using the 2100 Bioanalyzer System.

### RNA-Seq data analysis

RNA-seq data were trimmed and aligned to mouse genome (mm9), then Hisat2 42 was used to mapping the reads with default parameters. DEseq2 was used to calculate the significant different genes under the parameters: adjust of p value < 0.05 and log_2_ fold change > 1.

### ChIP-Seq and MeDIP-seq data analysis

ChIP-Seq and MeDIP-seq analysis was performed as described previously with minor revision 5, 43. Briefly, raw reads were trimmed to remove adaptor sequences and low-quality reads. Then bowtie2 tools were used to align the reads to mouse genome (mm9) with default parameters 44. After mapping, PCR duplicates were removed with samtools 45. MACS2 was used for predicting transcription factor peaks (q-value ≤0.01). Genome coverage files (bedgraph files) for visualization were generated by deepTools2 46. Histone modification and RNA polymerase II (PoI II) ChIP-seq data were obtained from our previous publication 12.

### Hi‐C data processing and normalization

Hi-C data was analyzed as previously described with minor revision 47 . Briefly, Hi-C clean sequencing reads were processed using the HiC-Pro 48. The reads were mapped against the mouse genome (mm9), and experimental artifacts, such as circularized reads and re‐ligations, were filtered out, and duplicate reads were removed. Aligned Hi‐C data were normalized using iterative correction (ICE) method 49 and visualized with Juicer tools v0.7.5 50. Using binned Hi‐C data, we generated 500 kb, 40 kb, 20 kb resolutions normalized Hi‐C matrices.

### Intra‐chromosomal contact frequency analysis

We plotted the frequency of cis‐chromosomal contacts in the normalized data at various genomic distances. By binning all cis‐chromosomal contact distances, frequency density was calculated with log_10_ distances).

### Compartment analysis

The compartment signal was computed as the first principle component (PC1) of R package (HiTC) 51 using 500kb normalized matrices with default parameters. A positive value indicates the A compartment and negative value is B compartment.

### TAD calling

TAD was identified by Domain Index (DI) method as previously described 52. Briefly, DI value was calculated based on the normalized matrix under 40kb bins with Hidden Markov Model (HMM). TAD boundaries were defined as the region <400 kb.

### TAD Compartment Switching

TAD was identified by Domain Index method. According to the compartment PC1 score, the mean value of each bin located in TAD was defined as TAD compartment score. In which, a positive value represented TAD in A compartment, while negative value was TAD in B compartment.

### Gene ontology (GO) analysis

GO enrichment was analysis using the Gene ontology website and chose an FDR (Benjamini-corrected p value) of less than 0.05.

### Statistical analysis

Results are presented as the mean ± SEM. Comparisons between two groups were performed using Student's t test for unpaired data. A p-value of <0.05 was considered significant.

## Results

### Characterization of female germline stem cells

**Figure 1:**
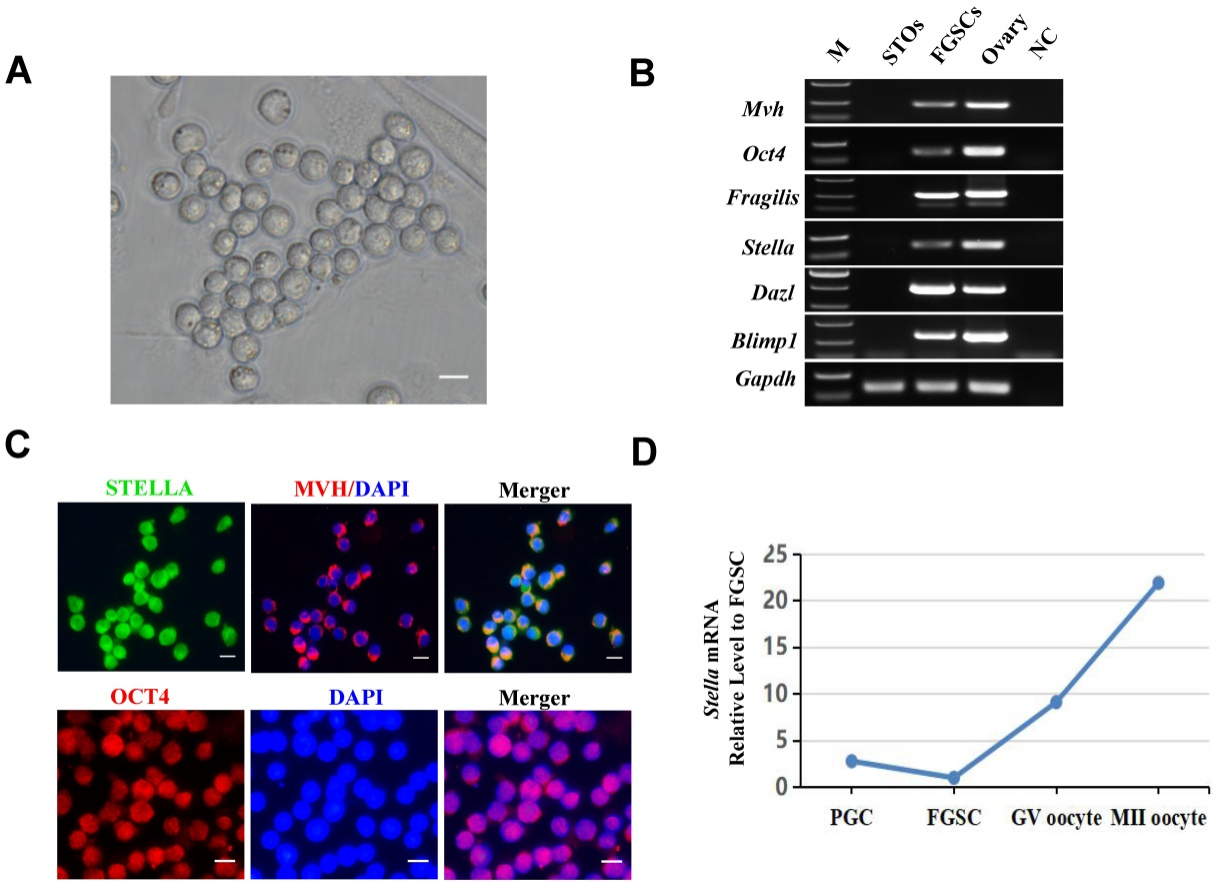
**Characterization of FGSCs.** (**A**) Brightfield image showing the morphology of FGSCs. (**B**) RT-PCR determination of *Mvh*,* Oct4*,* Fragilis*,* Stella*,* Dazl*, and* Blimp1* mRNA expression in FGSCs. *Gapdh* mRNA served as a control. M, DNA size markers. NC, negative control. (**C**) Immunofluorescence analysis of FGSCs with antibodies against MVH, STELLA, and OCT4. (**D**) The relative expression of *Stella* mRNA during FGSC development (Ma et al., 2019). Scale bar: 10 µm.

As shown in Figure 1A, the FGSCs used for this study were previously established in our laboratory 7. RT-PCR and immunofluorescence assays were performed to characterize the FGSCs. The expression of *Mvh* (also known as *Ddx4*),* Oct4*,* Fragilis*,* Stella*,* Dazl*, and* Blimp1* was determined by RT-PCR (Figure 1B). Immunofluorescence analysis showed positivity for STELLA, MVH, and OCT4 proteins (Figure 1C). In addition, *Stella* mRNA expression level gradually increased with female germ cell development (Figure 1D).

### *Stella* regulates proliferation and differentiation of female germline stem cells

**Figure 2:**
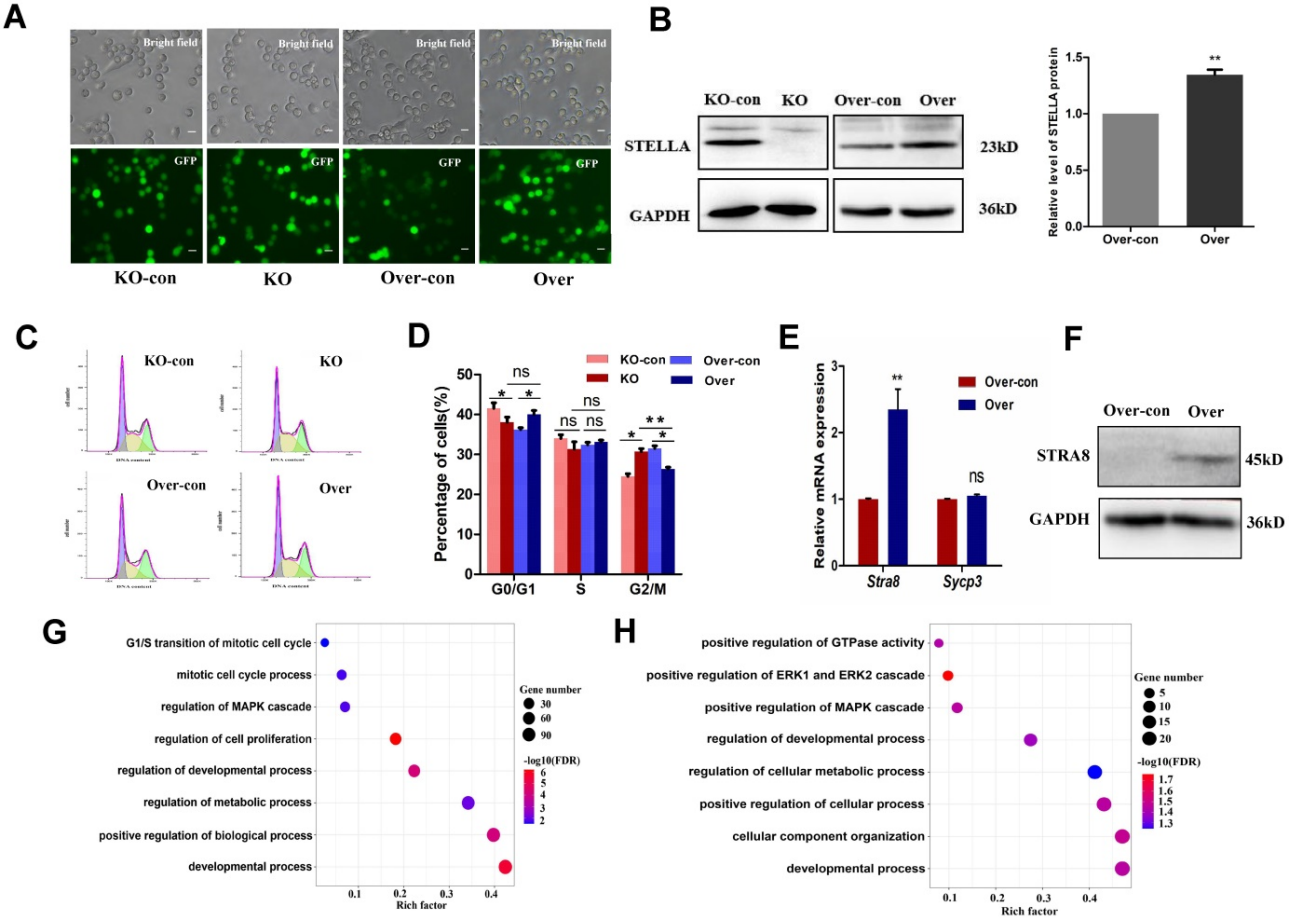
***Stella* affects self-renewal and differentiation of FGSCs.** (**A**) Fluorescence and bright field imaging of FGSCs infected with lentivirus. (**B**) A representative Western blot images (left) and quantitative analysis of overexpression group (right) is shown. (**C, D**) Cell cycle distribution profiles of FGSCs after *Stella* overexpression and knockout. (**E**) Relative mRNA expression of *Stra8* and* Sycp3* in FGSCs infected with the *Stella*-overexpressing lentivirus. (**F**) Relative protein expression of *Stra8* in FGSCs infected with the *Stella*-overexpressing lentivirus. (**G, H**) GO analysis of DEGs in *Stella*-knockout (**G**) and -overexpressing FGSCs (**H**) compared with the corresponding controls. KO-con, *Stella* knockout control. KO, *Stella* knockout. Over-con, *Stella*-overexpressing control. Over, *Stella* overexpression. Single asterisk represents p<0.05; while double asterisk indicates p<0.01. Scale bar: 10 µm.

To study the role of the *Stella* gene in FGSC development, CRISPR/Cas9 genome editing was used to knock out *Stella* in FGSCs (Figure 2A and Figure S1A). Two independent homozygous knockout FGSC lines were confirmed by genomic PCR, RT-PCR, and Sanger sequencing (Figure S1B-D). One knockout clone was randomly selected for the subsequent experiments. Western blotting was conducted to further verify the results (Figure 2B). Off-target analysis was carried out for each sgRNA, and the results indicated that no off-target mutations were found in any off-target sites (Figure S2A-D). Monoclonal FGSCs derived from *Stella* knockout could be maintained for more than 3 months until cryopreservation frozen *in vitro* (over passage 30). *Stella*-overexpressing FGSCs could be cultured *in vitro* for at least 4 months (over passage 30). Both *Stella* knockout and *Stella*-overexpressing FGSCs were passaged for at least 4 generations for further experiments (5-10 generations selected in this study).

Next, we examined the effect of *Stella* knockout on cell phenotype. The results showed that such knockout promoted FGSC proliferation, while *Stella* overexpression inhibited FGSC proliferation, based on CCK-8 and EdU assays (Figure S3A-C). This was consistent with the expression of proliferation-related genes *Etv5*, *Bcl6b*, *Oct4*, and *Akt* in *Stella-*knockout and -overexpressing FGSCs (Figure S3D). Since cell proliferation is closely associated with cell cycle progression 53, we investigated the potential effects of *Stella* on the cell cycle. Cell cycle analysis showed that *Stella* knockout increased the proportion of cells in G_2_/M phase and decreased that in G_0_/G_1_ phase (Figure 2C-D). Meanwhile, *Stella* overexpression showed the opposite results (Figure 2C-D). Moreover, *Stra8* mRNA expression was upregulated after *Stella* overexpression rather than *Stella* knockout (Figure 2E), which is consistent with WB results (Figure 2F).

To gain further insight into the mechanism by which *Stella* regulates FGSC development, we obtained transcriptional profiles by RNA sequencing (RNA-seq). Principal component analysis (PCA) showed that three repeats of each sample clustered together (Figure S4A). The RNA-seq results were also confirmed by RT-qPCR for selected genes (Figure S4B), indicating that the RNA-seq data were reliable. A heatmap of differentially expressed genes (DEGs) showed clear differences upon *Stella* knockout and *Stella* overexpression (Figure S4C-D). Gene Ontology (GO) terms of the DEGs between *Stella*-knockout and control groups were related to G_1_/S transition of the mitotic cell cycle, regulation of cell proliferation, and developmental process (Figure 2G). Furthermore, KEGG pathway analysis of the DEGs between *Stella-*knockout and control groups showed associations with the NF-kappa B signaling pathway, JAK-STAT signaling pathway, and PI3K-AKT signaling pathway (Figure S4E-F). As previously reported, the PI3K-AKT signaling pathway is involved in regulating FGSCs 5. In addition, GO terms associated with *Stella* overexpression included biological processes related to the developmental process, positive regulation of cellular process, and regulation of cellular metabolic process (Figure 2H). In brief, these results suggest that *Stella* knockout enhanced FGSC proliferation or self-renewal, whereas *Stella* overexpression inhibited FGSC growth and promoted cell differentiation.

### *Stella* modulates genome-wide DNA methylation patterns in female germline stem cells

**Figure 3:**
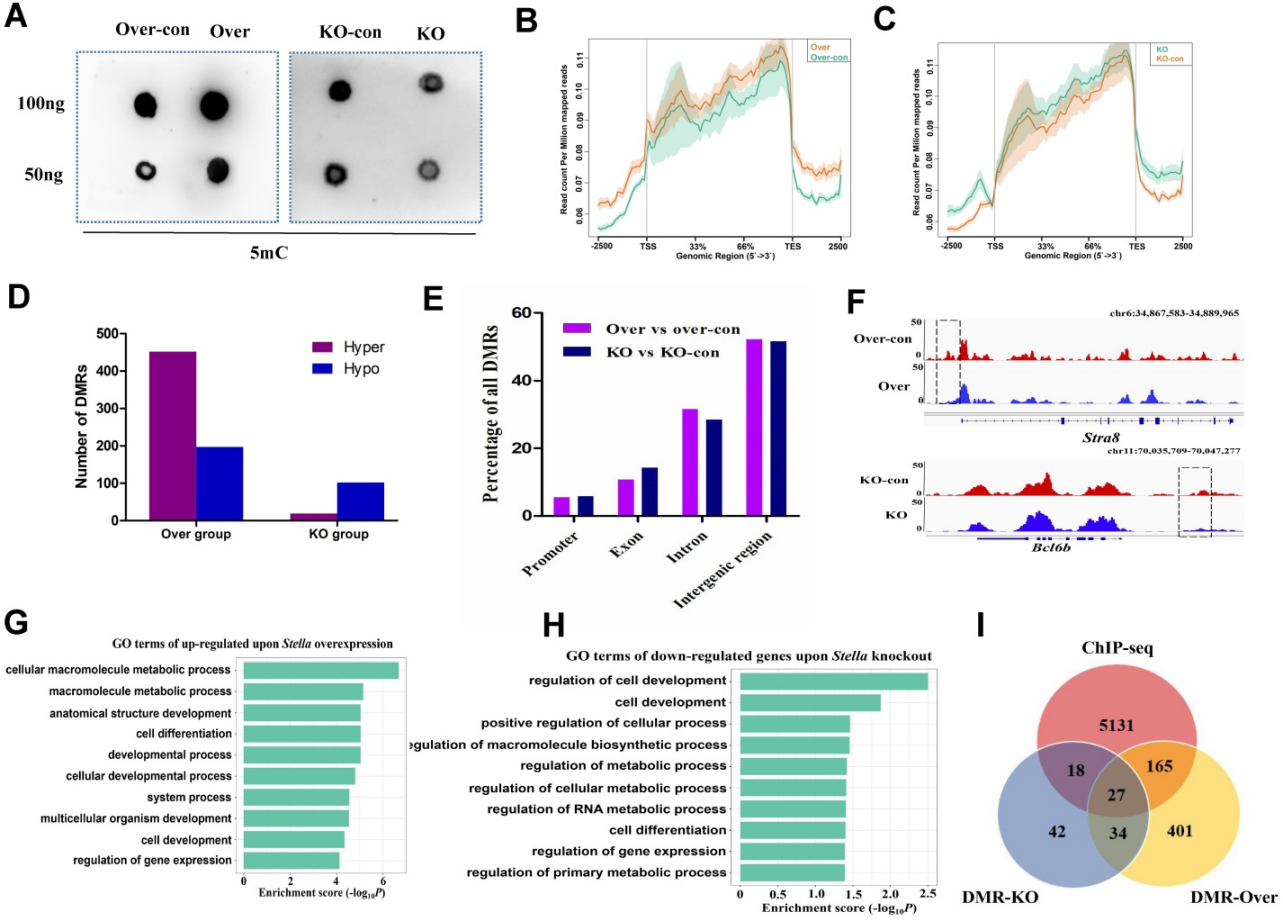
**Effect of *Stella* on genome-wide DNA methylation**. (**A**) Dot blotting of DNA methylation. (**B**,** C**) Methylation distribution of gene body and flanking regions in *Stella*-overexpressing (**B**) and -knockout FGSCs (**C**). (**D**) Histograms showing the numbers of DMRs between the Over group (over vs over-con), or KO group (KO vs KO-con). (**E**) Distribution of DMRs in different genomic features. (**F**) A snapshot of the IGV genome browser showing DNA methylation (5mC) signal at the *Stra8* locus (top track) and *Bcl6b* locus (bottom track). (**G, H**) GO enrichment analysis of upregulated DEGs upon *Stella* overexpression (**G**) and downregulated DEGs upon *Stella* knockdown (**H**). (**I**) Venn diagrams showing the number of STELLA ChIP-seq peaks overlapping with DMRs. KO-con, *Stella* knockout control. KO, *Stella* knockout. Over-con, *Stella*-overexpressing control. Over, *Stella* overexpression. DMR-KO, DMR genes number upon *Stella* knockout. DMR-Over, DMR genes number upon *Stella* overexpression.

To explore molecular mechanism of *Stella* in FGSC development, we performed dot blotting assays. The results showed that DNA methylation was positively correlated with *Stella* expression level in FGSCs (Figure 3A), indicating *Stella* is related to DNA methylation in FGSCs. Then, we performed methylated DNA immunoprecipitation (MeDIP) to examine the effect of *Stella* on genome-wide DNA methylation levels in FGSCs. The methylation profiles showed a clear trend, low DNA methylation around transcription start sites (TSSs) and higher DNA methylation around transcription end sites (TESs) (Figure 3B-C). We detected 649 differentially methylated regions (DMRs) (452 hypermethylated and 197 hypomethylated) between the overexpression group, and 121 DMRs (19 hypermethylated and 102 hypomethylated) between the knockout group, respectively (Figure 3D). These findings were consistent with the results of dot blotting (Figure 3A), where *Stella* overexpression increased the level of DNA methylation, and *Stella* knockout reduced it. After mapping DMRs to the nearest genomic features, we found that the majority of DMRs were located in intergenic and intronic regions, and only a small portion of DMRs were identified in gene promoters (Figure 3E), suggesting DMRs were unevenly distributed across the genome. DNA demethylation at the gene promoter region usually upregulates gene expression 54. The analysis results demonstrated that *Stra8* gene promoter region was demethylated after *Stella* overexpression (Figure 3F), which was consistent with the upregulation trend of *Stra8* expression (Figure 2E-F). Similarly, demethylation of* Bcl6b* gene promoter after *Stella* knockout was accompanied by upregulation of *Bcl6b* mRNA expression (Figure 3F, S3D). GO terms particularly associated with genes upregulated upon *Stella* overexpression were cell differentiation, cell development, and regulation of gene expression (Figure 3G). Meanwhile, GO terms particularly associated with genes downregulated upon *Stella* knockout were regulation of cell development, positive regulation of cellular process, and regulation of gene expression (Figure 3H). We also compared STELLA binding sites with the DMRs upon *Stella* overexpression and *-*knockout. The results showed that 37% of *Stella-*knockout DMRs and 31% of* Stella*-overexpression DMRs overlapped with STELLA binding sites at the genome-wide level (Figure 3I). Moreover, the ChIP-seq of STELLA was highly correlated with the DNA methylation (Figure S5A-B). Thus, above findings indicated that a subset of DNA methylation regions was directly regulated by STELLA binding. Overall, these results suggest that *Stella* regulates FGSC development via DNA methylation.

### STELLA and active histone modification are enriched near TAD boundaries

**Figure 4:**
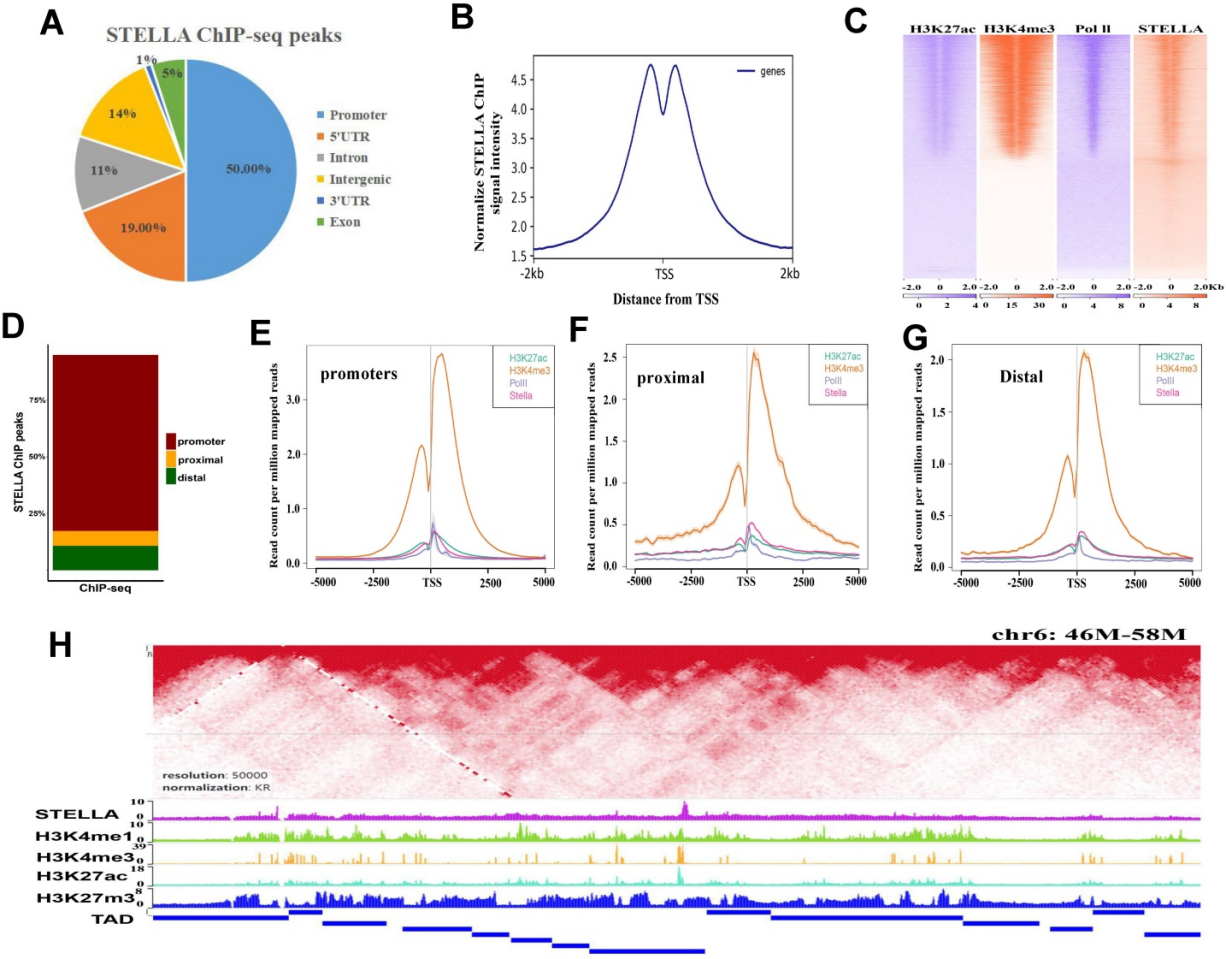
** STELLA and active histone modifications are enriched near TAD boundaries.** (**A**) Pie chart representing the distribution of STELLA ChIP-seq peaks relative to genes. (**B**) The distance from the TSS to the promoter targets for STELLA is plotted. (**C**) Heatmap of *Stella* and histone marks from previously published datasets H3K4me3, H3K27ac, and Pol II ChIP-seq (±2.5 kb from peak center). (**D**) Bar plot shows percent enrichment of STELLA ChIP-seq peaks at distal, promoter, and proximal regions. (**E-G**) Density plot of ChIP-seq signals of STELLA and histone marks centered at STELLA peak located in distal, proximal, and promoter regions. (**H**) Heatmap showing that STELLA and active histone modifications are enriched near TAD boundaries.

To identify genomic loci occupied by STELLA proteins, we performed STELLA ChIP-seq experiments. Annotation of ChIP-seq peaks revealed that 50% of STELLA binding sites were localized in promoter regions and 25% of the binding sites were located within introns and intergenic regions (Figure 4A). Consistent with the genome annotation, the normalized STELLA binding signal was high at promoter regions (Figure 4B). Furthermore, we found that the STELLA ChIP correlated well with previously published histone modification data (Figure 4C). We further categorized STELLA peaks into three classes based on the distance from TSSs (Figure 4D). The enrichment of STELLA at promoter, proximal, and distal sites showed clear overlap with active TSS marks (H3K27ac, Pol II, and H3K4me3) (Figure 4E-G), which implied that *Stella* is likely to be involved in gene expression regulation. Moreover, integrative analysis of ChIP-Seq and published Hi-C data 9 showed that the binding sites of STELLA and active histones H3K4me3 and H3K27ac were enriched near the TAD boundaries (Figure 4H), suggesting STELLA is related to chromatin structure remodeling.

### *Stella* overexpression perturbs chromatin interactions

**Figure 5:**
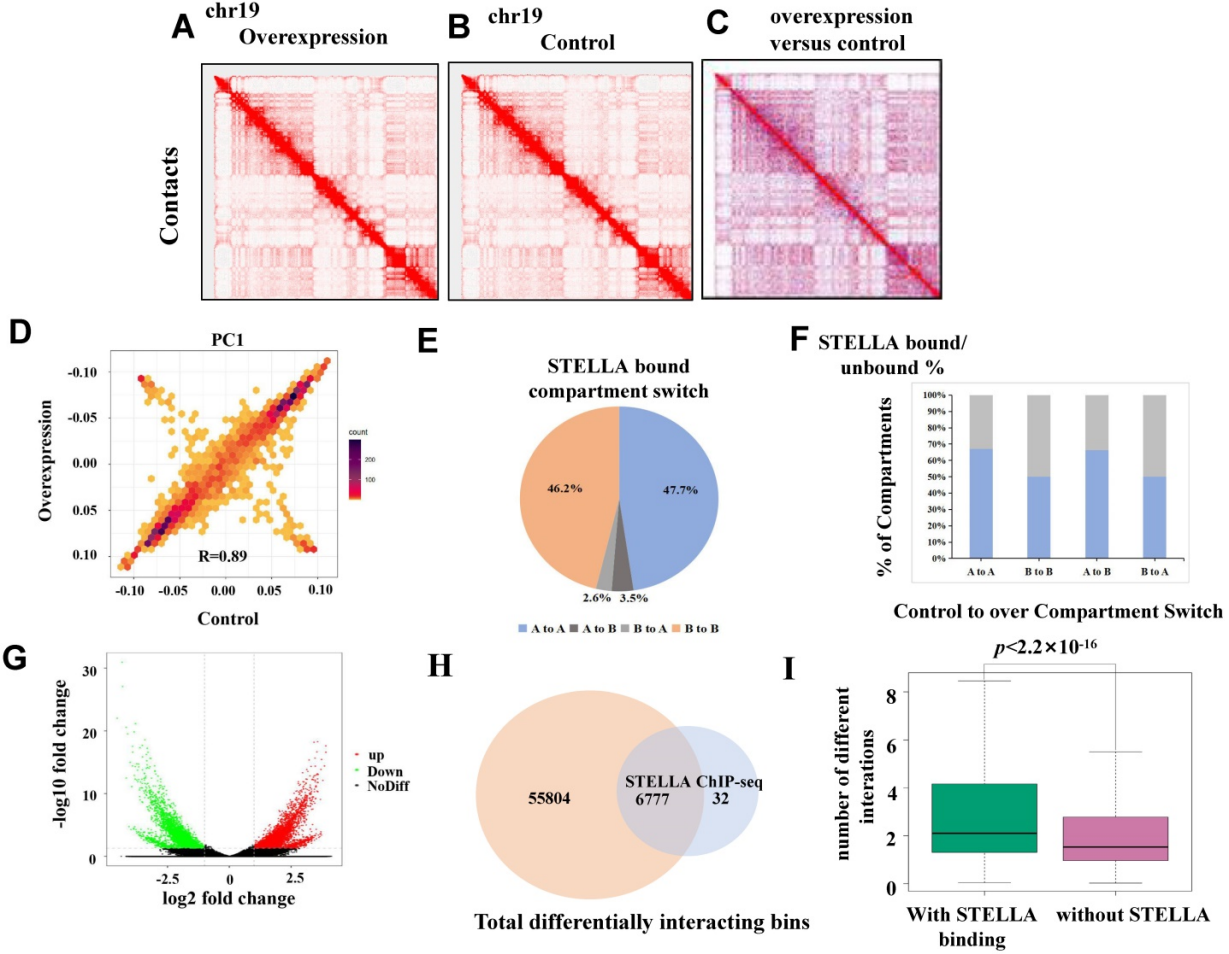
**Stella overexpression perturbs chromatin interactions.** (A) Genome-wide chromatin conformation Hi-C analysis was performed using two replicates of Stella-overexpressing and controls. Representative normalized Hi-C interaction heatmaps of chromosome 19 at 1 Mb resolution are shown in (A) Stella-overexpressing and (B) control cells. (C) Differential interaction heatmap for chromosome 19 (1 Mb), showing bins for upregulated (red) and downregulated (blue) interactions. (D) The correlation of cis-eigenvector 1 values between the Stella-overexpressing and control cells. Correlation coefficient (r)=0.89. (E) Pie chart showing the compartment-switching profiles of STELLA-bound regions. (F) Bar graph showing the percentage of the compartment-switching regions that are bound by STELLA. The colored portions of the graph denote the STELLA-bound percentage of each compartment-switching category. (G) Volcano plot representing differential interactions (DI) at 20 kb resolution. (H) Venn diagrams showing the number of STELLA ChIP-seq peaks overlapping with all differential interacting bins at 20-kb resolution. (I) Plot showing number of differential interactions calculated in 20-kb bins, based on the presence and absence of overlapping STELLA peaks at genome wide level.

To further study the effect of *Stella* on chromatin structure, we performed *in situ* Hi-C experiments with *Stella-*overexpressing FGSCs and the control groups. More than 700 million total reads were obtained (Table S4). At the chromosomal scale, Hi-C contact maps appeared similar between the *Stella-*overexpressing group and the control (Figure 5A-C). The PC1 values were also highly correlated (Figure 5D, R=0.89). Consistent with this, the majority of the compartmentalization was largely unchanged upon *Stella* overexpression (Figure S6A). Upon *Stella* overexpression, only 3.4% of the genome had a transition from compartment A-type to compartment B-type, while 2.8% showed a transition in the opposite direction (Figure S6B). Next, we investigated whether the STELLA binding regions were associated with compartment switching. Among all STELLA-bound sites, 47.2% of peaks were located within the open compartment A regions, 46.2% of peaks were located within the closed compartment B regions, and ~7.1% of STELLA peaks were found in regions showing compartment-switching (Figure 5E). We assessed whether the percentage of genomic compartment-switching regions was related to a state of being either bound or unbound by STELLA. The proportion of STELLA binding sites is 75% of “A to B” compartment-switching regions, which was similar to 55% of “B to A” (Figure 5F). This suggests that *Stella* is unlikely to mediate compartment switching.

We compared Hi-C data between the *Stella*-overexpressing and control groups to obtain a deeper insight into the differential interaction. The results revealed differences in genome-wide chromatin interactions between *Stella*-overexpressing and control groups (Figure 5G). Furthermore; we found that 99.5% of STELLA binding sites overlapped with differentially interacting regions at the genome-wide level (Figure 5H). After calculating the number of differential interactions within or without STELLA enrichment, we observed that the number of differential interactions involving STELLA binding sites was significantly higher than that without STELLA binding (Figure 5I). Overall, the results suggest that *Stella* regulates chromatin interactions, while not affecting compartment formation.

### *Stella* overexpression enhances the TAD boundary strength in STELLA-associated regions

**Figure 6:**
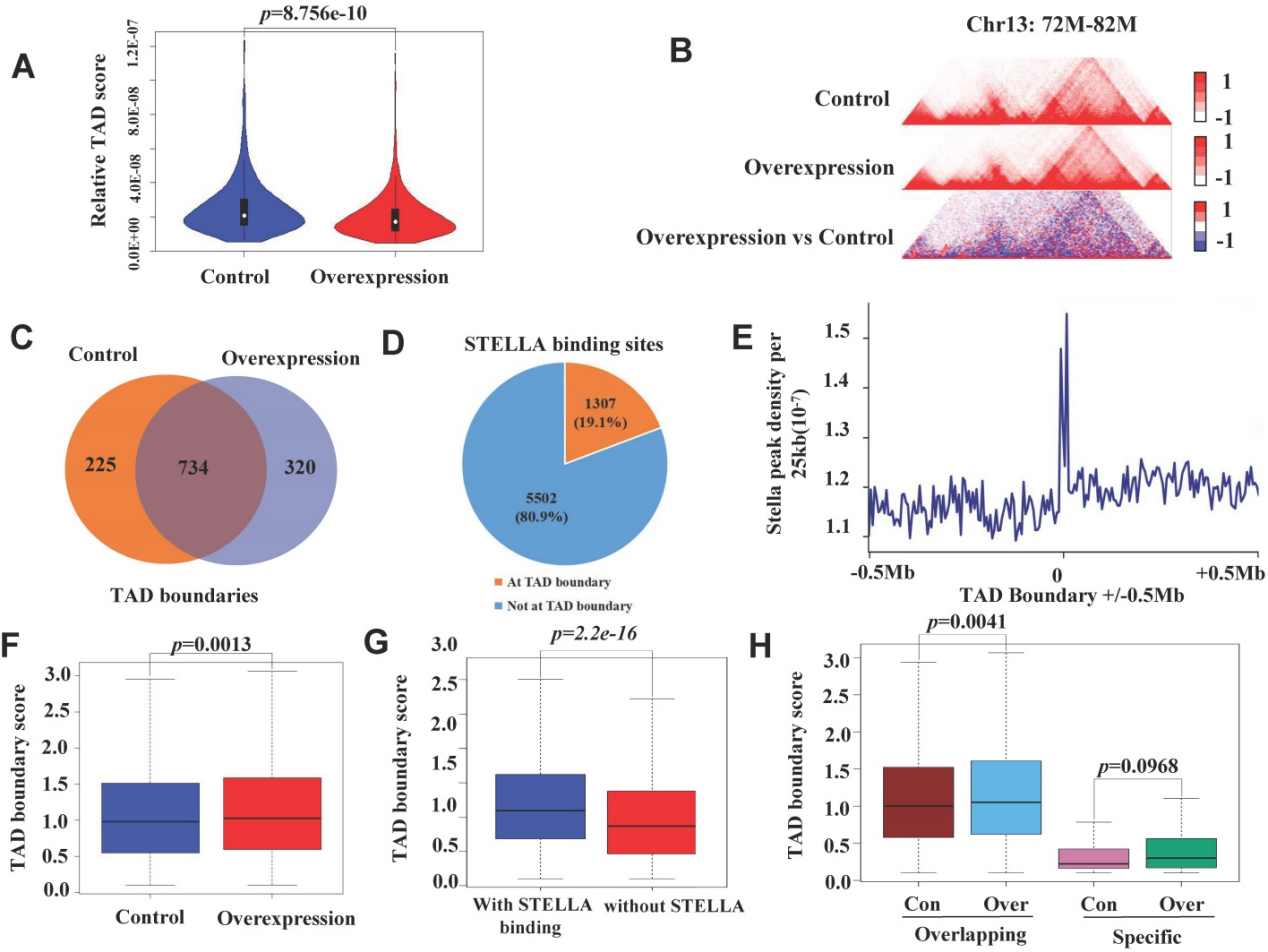
***Stella* overexpression enhances the TAD boundary strength in STELLA-associated regions.** (**A**) Genome-wide statistical analysis within TADs at 400 kb resolution. (**B**) A representative region showing contacts and TAD boundaries at 20 kb resolution. (**C**) Venn diagram showing the number of overlapping TAD boundaries between control and *Stella*-overexpressing cells. (**D**) Pie chart showing the percentage of STELLA localization at TAD boundaries. (**E**) The frequency plot of STELLA ChIP-seq peaks per 25 kb for ±0.5 Mb of each overexpression TAD boundary. (**F**) Box plot showing that the TAD boundary intensity score was higher after *Stella* overexpression. (**G**) STELLA binding is associated with higher TAD boundary scores. Box plot showing the TAD boundary scores for STELLA-bound and unbound TAD boundaries. (**H**) Box plot showing the TAD boundary score distribution for the overlapping and control and *Stella*-overexpressing cell-specific TAD boundaries.

To examine the effect of *Stella* on TAD organization, we first identified 1740 and 1723 TADs in STELLA-overexpressing and control groups (Figure S7A), respectively. Most of the TADs overlapped between STELLA-overexpressing and control groups (Figure S7B). Consistent with this, we found that the size of TADs was similar between *Stella*-overexpressing and control groups (Figure S7C). We also assessed the distribution of TADs within A/B compartments and found that about 4.6% of them switched at the genome-wide level (Figure S7D). Nevertheless, genome-wide statistical analysis showed the interaction within TADs reduced after *Stella*-overexpressing (Figure 6A-B).

In addition, we identified 1054 and 959 TAD boundaries in *Stella*-overexpressing and control groups, respectively (Figure 6C). Although TADs are stable across different cell types and species 52, 55, *Stella* overexpression still altered the localization of ∼23% of TAD boundaries (Figure 6C). An analysis of the overlap of STELLA ChIP-seq peaks with TAD boundaries revealed that ∼19% of STELLA binding sites were located at TAD boundaries (Figure 6D). We also observed the enrichment of STELLA binding at the TAD boundaries (Figure 6E). Next, we wondered whether *Stella* regulates TAD boundary strength. The analysis showed that the overall TAD boundary strength increased after *Stella* overexpression (Figure 6F). Furthermore, integrative analysis of Hi-C and ChIP-seq showed the TAD boundary strength was higher in STELLA binding sites than unbinding region (Figure 6G). Interestingly, *Stella* overexpression resulted in an overall increase in the overlapping TAD boundaries not the specific TAD boundaries (Figure 6H). Consistently, we next examined TADs in *Stella*-knockout FGSCs. A total of 1951 TADs was identified in *Stella*-knockout FGSCs, most of which overlapped with the control groups (Figure S8A). In addition, genome-wide statistical analysis showed the interaction within TADs enhanced (Figure S8B-C), while the overall TAD boundary strength reduced after *Stella* knockout (Figure S8D). Unlike the case with overexpression, *Stella* knockout weakened the TAD boundary strength of overlapping and specific regions (Figure S8E). Taken together, these results demonstrate that *Stella* regulates TAD boundary strength in *Stella*-associated regions.

## Discussion

*Stella* plays significant roles in chromatin condensation and epigenetic modification 56-58. However, despite its importance, there has thus far been relatively little research on *Stella*, especially for germ cells *in vitro*. The results obtained in this work demonstrated that *Stella* regulates FGSC development at multiple epigenetic levels, including DNA methylation, chromatin interaction, and TAD boundary strength.

*Stella* was first discovered in mice when its expression was shown to occur in gastrulating embryos, which may represent the differentiation of a subpopulation of cells into the PGCs lineage 56. Our results showed that *Stella* knockout enhanced FGSC proliferation. Additionally, *Stella* overexpression in FGSCs was found to promote the expression of *Stra8*, which is an essential “gatekeeper” in the initiation of meiosis 59. The upregulation of its expression may indicate that the FGSCs had entered pre-meiosis.

A better understanding of the *Stella* gene would provide us with a new perspective on *in vitro* differentiation. To date, our understanding of the role of *Stella* in regulating DNA methylation has been obscured by conflicting results from different developmental stages or cell types 21, 26, 57. We found that genome-wide DNA methylation was reduced in FGSCs after *Stella* knockout. This trend corresponds to previously reported results showing that *Stella* protects DNA methylation from TET2 and TET3 enzyme-dependent oxidation 26. In parallel with this, the level of global DNA methylation was upregulated in FGSCs after *Stella* overexpression. This pattern is also coherent with oogenesis, during which the levels of DNA methylation and *Stella* expression increase 5. One possible explanation for the pattern of *Stella* on DNA methylation may be related to its subcellular localization 20, 60, 61.

In addition to DNA methylation and DNA demethylation 54, 62, 63, dramatic remodeling of the chromatin also occurred in the early germline 64-66. Although *Stella* overexpression does not alter Compartment A/B and TAD formation, what is surprising is that *Stella* overexpression reduce the interaction within TADs and enhance the boundary strength of TADs in STELLA-associated regions. These findings are consistent with previous observations that the interaction frequency within TADs decreased during oocyte maturation 67. Our findings also mean that *Stella* overexpression reduces the frequency of contacts between the target genes and regulatory elements. Given that the proper folding of chromatin is crucial for gene regulation, increasing attention has been drawn to the relationship between alterations in chromatin structure and diseases. Destruction of TAD boundaries would lead to the dysregulation of gene expression and diseases 68, 69. *Stella* knockout has been shown to lead to an abnormal heterochromatin distribution in fully grown GV oocytes 70, 71, subsequently impaired oocytes and embryo development 19.

In addition to *Stella*, FGSCs also express other germline genes such as *Ddx4*, *Fragilis*, and *Dazl* 2. Most of them have been reported to play critical roles in germ cell development. For example, the depletion of *Dazl* causes dysregulation of maternal transcripts during mouse oocyte maturation 72. Moreover, knockdown of *Ddx4* reduces the number of germ cells in the gonads of male and female embryos 73. Whether these germline genes regulate germ cell development by affecting chromatin structure warrants further study. Based on our combined findings, our study reveals not only the role of *Stella* in regulating DNA methylation, but also that it affects the 3D structure of chromatin. Our findings deepen our understanding of the epigenetic mechanisms of FGSC development. Further research should focus on how *Stella* coordinates the relationship between DNA methylation and 3D chromatin structure in FGSCs.

## Supplementary Material

Supplementary figures.

Supplementary table.
