# Supplementary material for: Stella Regulates the Development of Female Germline Stem Cells by Modulating Chromatin Structure and DNA Methylation
Source: Int J Biol Sci. 2022 Apr 18;18(7):3006–18. doi: 10.7150/ijbs.69240 (PMC9066111; doi:10.7150/ijbs.69240)
Supplement: Supplementary file 1 — Supplementary figures. [file ijbsv18p3006s1.pdf]

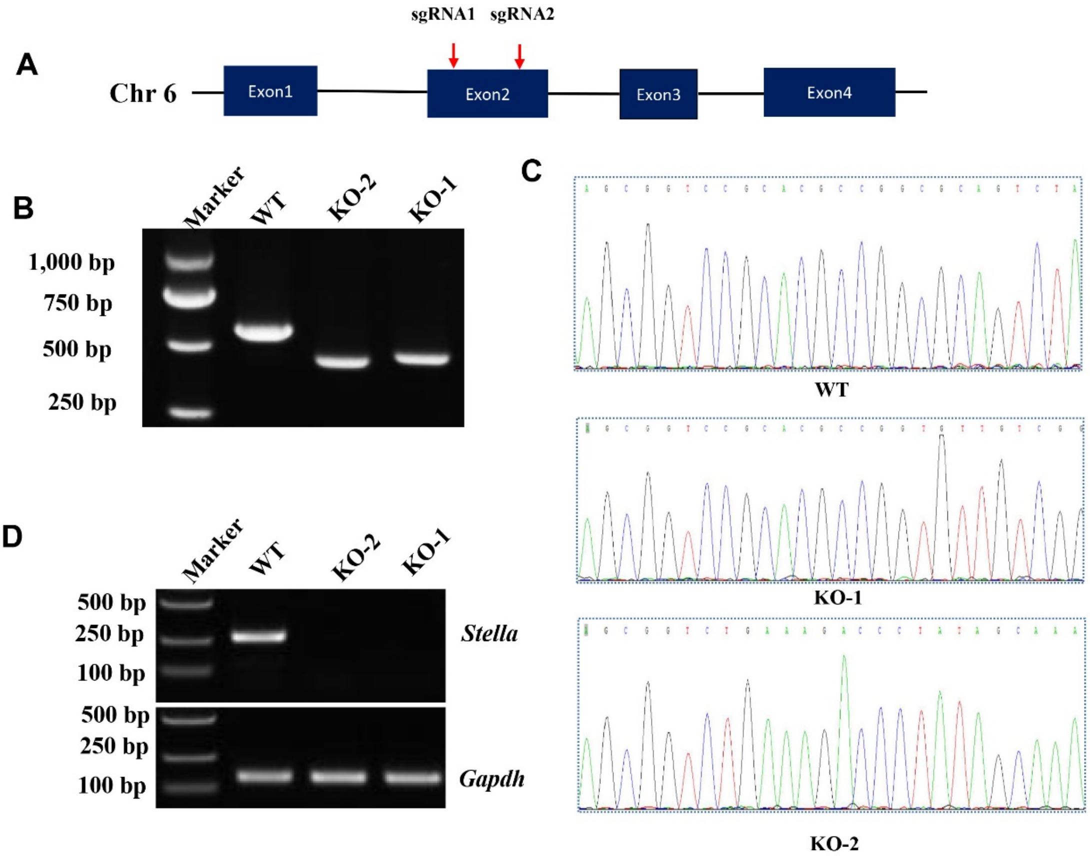

Figure S1. Generation of *Stella*-knockout cell lines by CRISPR/Cas9-mediated genome editing. (**A**) Schematic diagram of sgRNA targeting sites. (**B**) PCR analysis of genomic DNA. (**C**) Sanger sequencing validation studies. (**D**) RT-PCR analysis of the *Stella* mRNA level.

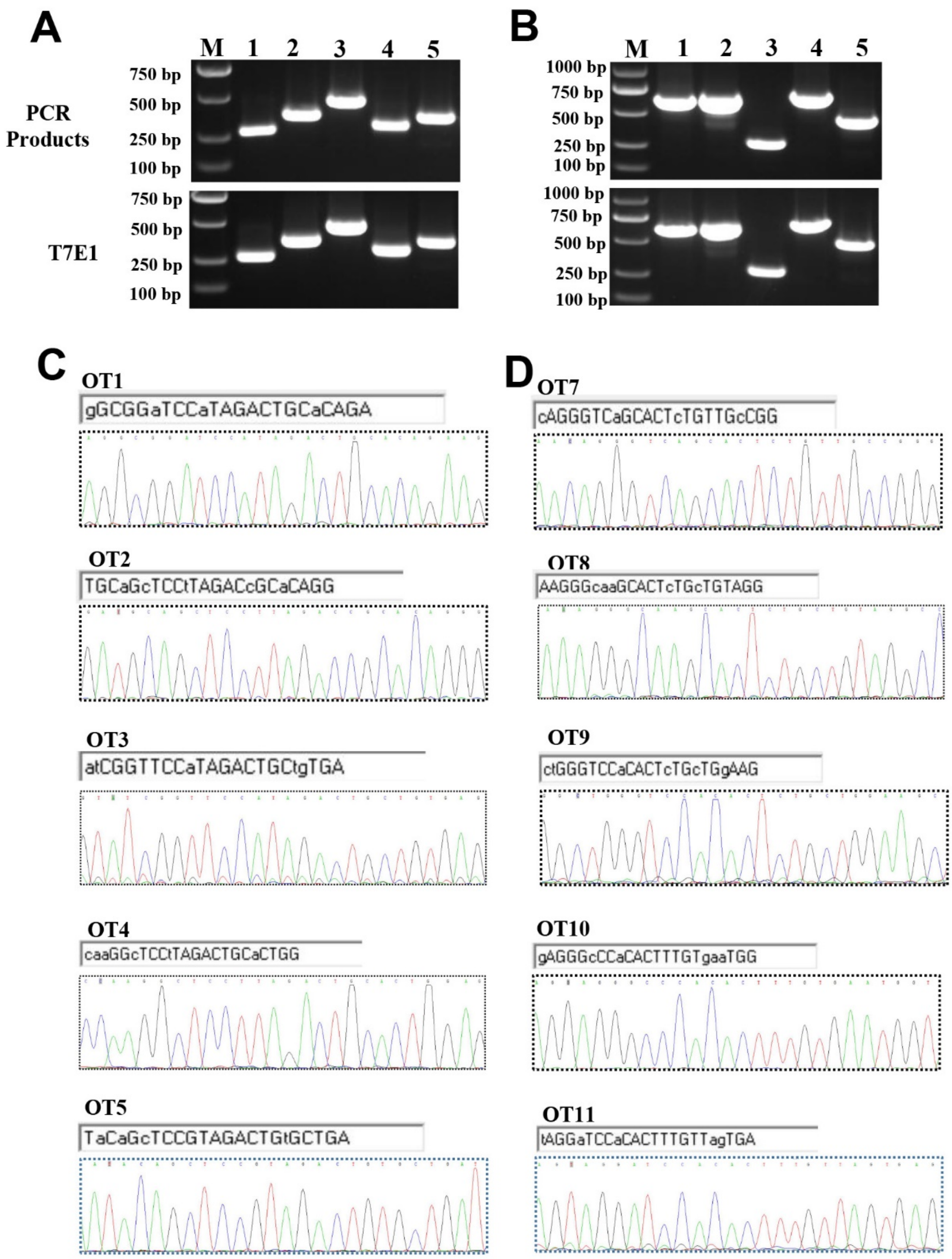

Figure S2. Analysis of potential off-target sites. (**A**) T7E1 cleavage analysis of potential off-target sites for sgRNA1. M, DL2000; 1–5 represent potential off-target sites. (**B**) T7E1 cleavage analysis of potential off-target sites for sgRNA2. M, DL2000; 1–5 represent potential off-target sites. (**C**) Five potential off-target sites for sgRNA1 of stella were sequenced. Twenty base pairs of POTS and PAM are represented in shadow. (**D**) Five potential off-target sites for sgRNA2 of stella were sequenced. Twenty base pairs of POTS and PAM are represented in shadow.

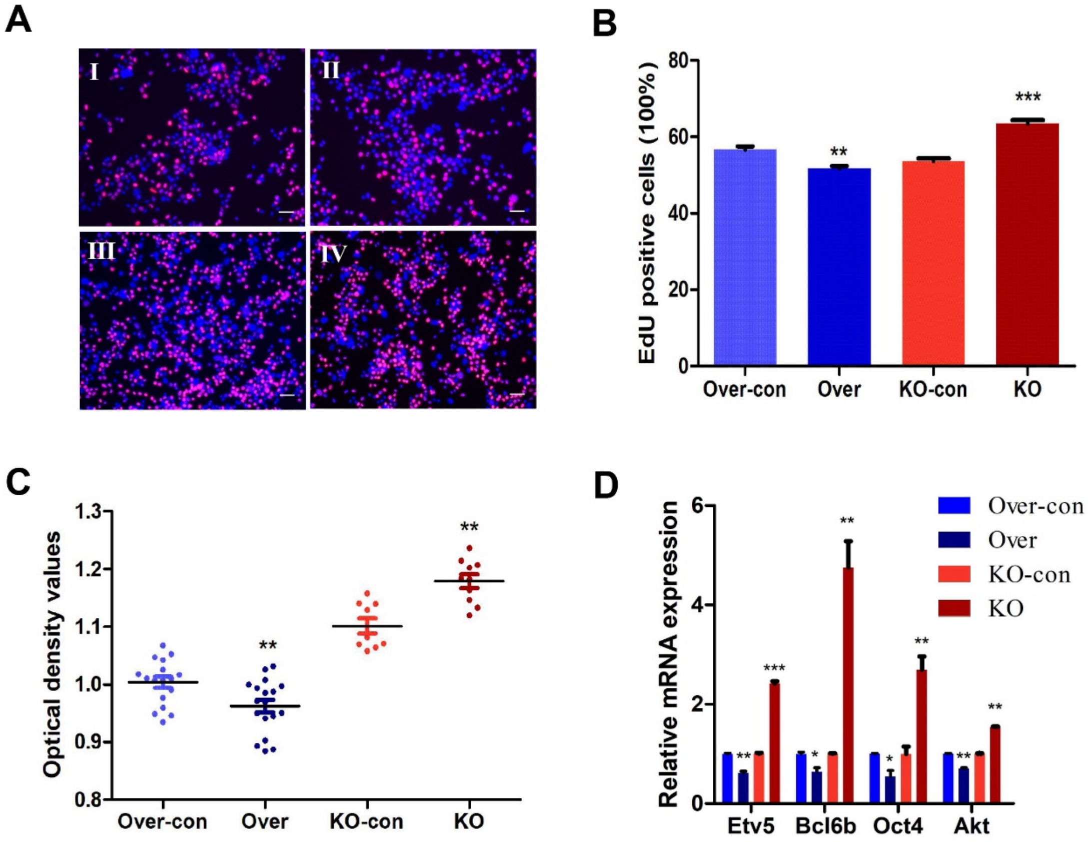

Figure S3. Cell proliferation experiment of FGSCs in vitro. (**A**) EdU staining assays. (**B**) Quantification of EdU assay results. (**C**) CCK-8 assay. (**D**) Relative expression of *Etv5, Bcl6b, Oct4, and Akt* in FGSCs after *Stella* overexpression and knockout. KO-con, *Stella*-knockout control. KO, *Stella* knockout. over-con, *Stella*-overexpressing control. over, *Stella* overexpression.

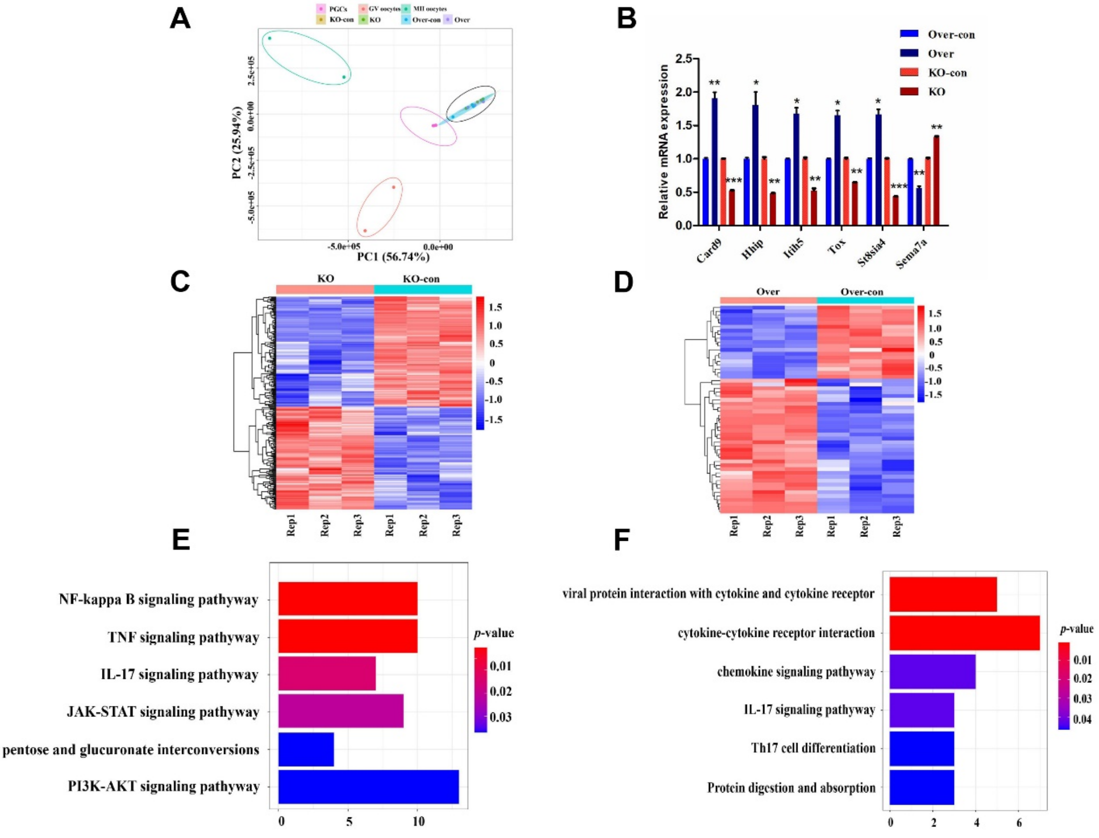

Figure S4. Differential gene expression analysis by RNA-seq. (**A**) Principal component analysis (PCA) of gene expression profile of PGCs, GV oocytes, MII oocytes, *Stella*-knockout and -overexpression FGSCs. (**B**) RNA-seq data were identified by randomly selected genes. (**C, D**) Heatmap of the differentially expressed genes between *Stella*-knockout FGSCs (**C**) and *Stella*-overexpressing FGSCs (**D**) compared with the corresponding controls for each biological replicate. (**E, F**) KEGG pathway terms of differentially expressed genes in *Stella*-knockdown (**E**) and *Stella*-overexpressing FGSCs (**F** ~~G~~). KO-con, *Stella*-knockout control. KO, *Stella* knockout. over-con, *Stella*-overexpressing control. over, *Stella* overexpression.

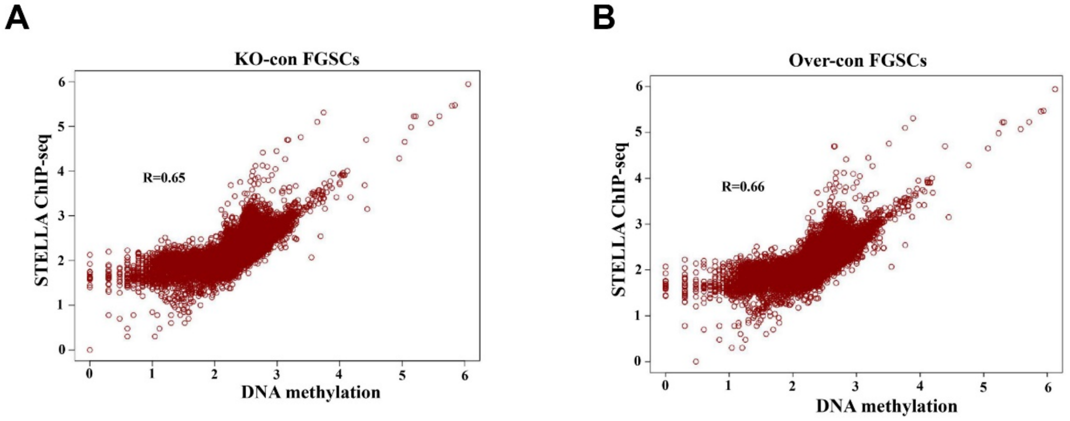

Figure S5. Scatter plots showing the correlation between DNA methylation and STELLA binding sites in FGSCs. The correlation between DNA methylation and ChIP-seq of STELLA was analyzed in KO-con FGSCs (**A**) and KO-con FGSCs (**B**), respectively. KO-con, *Stella*-knockout control. Over-con, *Stella*-overexpressing control.

A

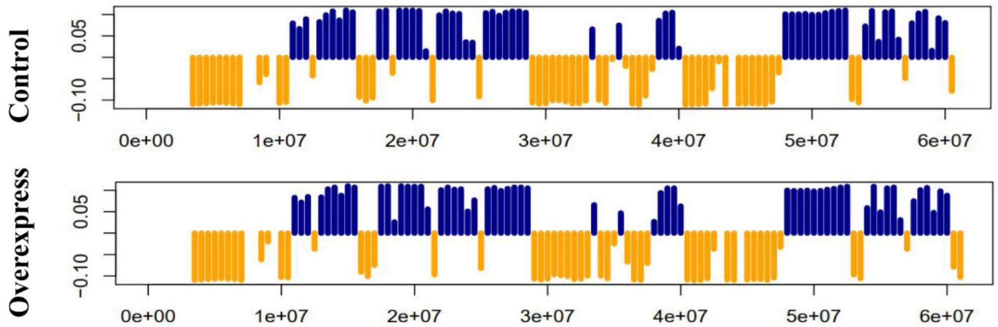

B

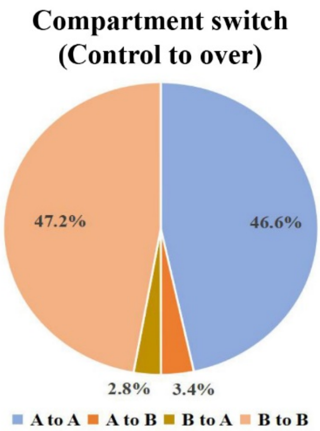

Figure S6. *Stella* overexpression does not affect the formation of compartments. (**A**) Tracks of cis-eigenvector 1 values across the entirety of chromosome 19 are very similar between the *Stella*-overexpressing and control cells. (**B**) Pie chart showing the genomic compartment changes between *Stella*-overexpressing and control cells. Data sets "A" and "B" denote the open and closed compartments, respectively. "A to A" represents compartments that are open in both cell lines; "B to B" represents compartments that are closed in both cell lines; "A to B" denotes compartments that are open in control but closed in *Stella*-overexpressing cells; and "B to A" denotes compartments that are closed in control and open in *Stella*-overexpressing cells.

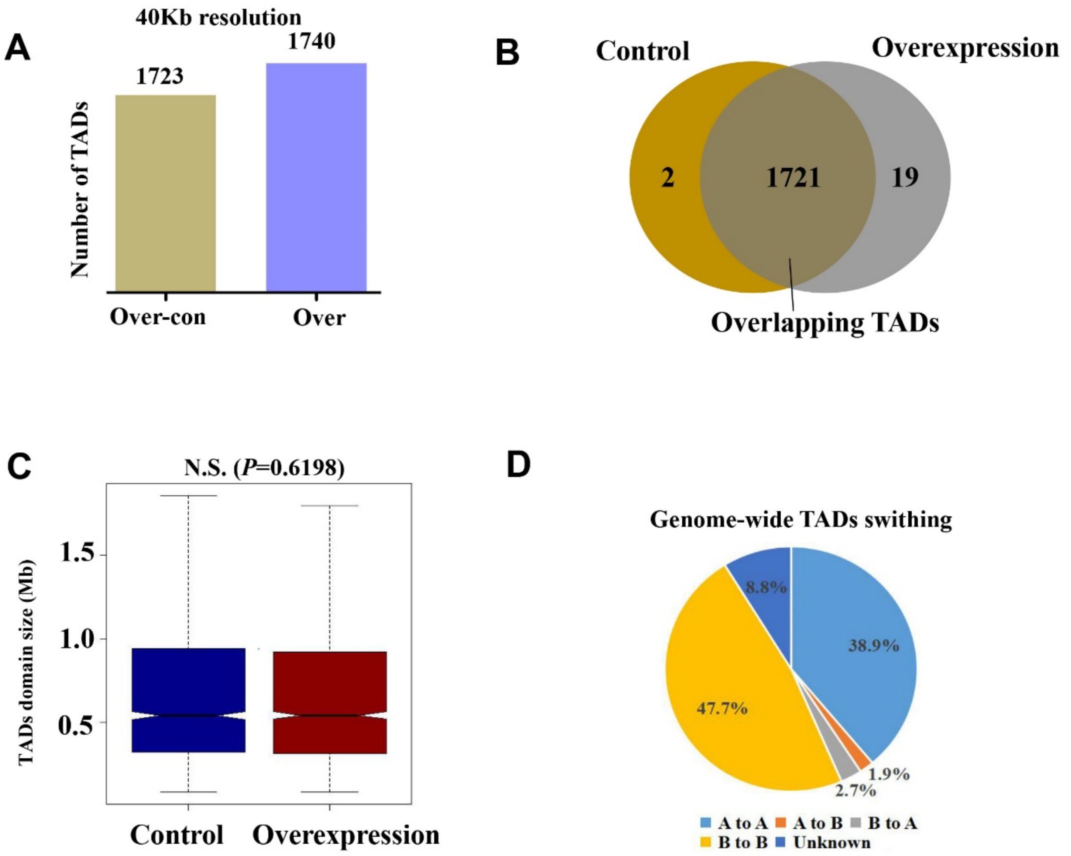

Figure S7. *Stella* overexpression does not affect the formation of TADs. (**A**) Number of TADs identified in control and *Stella*-overexpressing groups at 40 kb resolution. (**B**) Venn diagram showing that the numbers of TADs are largely similar between *Stella*-overexpressing and control groups. (**C**) Box plot showing the average size (kb) of TADs identified in control and Stella-overexpressing groups, both at 40 kb (t-test, p-value=0.6198). (**D**) Pie chart showing TAD switching between A/B compartments. "A to A" and "B to B" mean no switching of TADs; they remain in the same "A" and "B" compartments, respectively. "A to B" represents TADs switching from A-type in the control to B-type, and "B to A" represents TADs switching from B-type in the control to A-type in *Stella*-overexpressing groups. Unknown represents when the compartment status of TADs is not known.

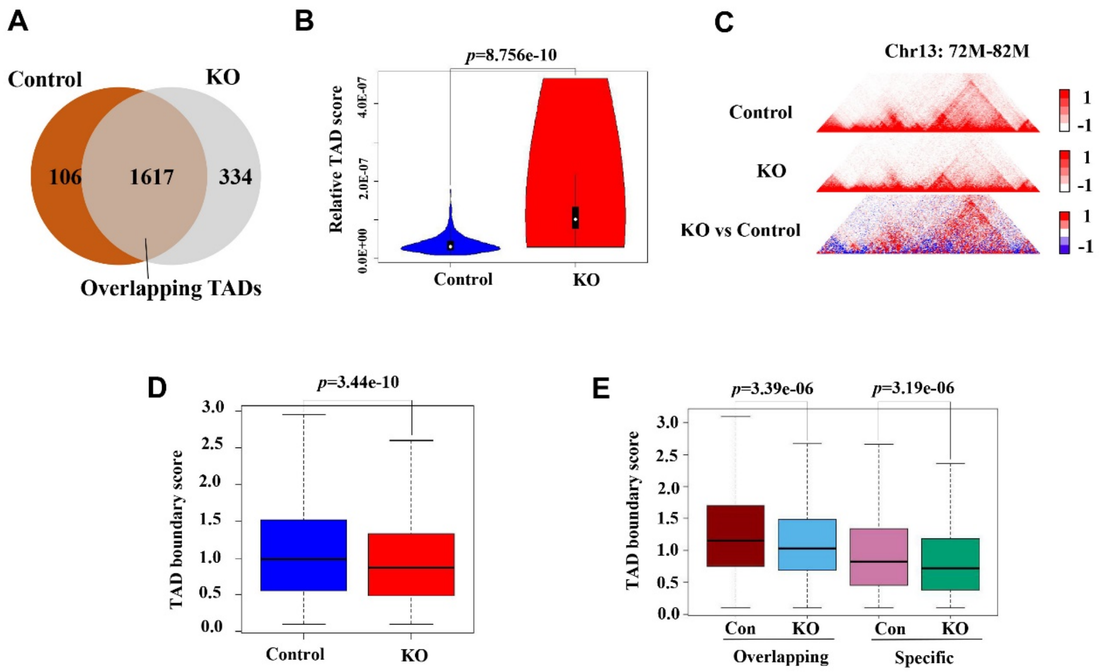

Figure S8. *Stella* knockout reduces the TAD boundary strength in STELLA-associated regions. (**A**) Venn diagram showing that the numbers of TADs are largely similar between *Ste*lla knockout and control groups. (B) Genome-wide statistical analysis within TADs at 400 kb resolution. (C) A representative region showing contacts and TAD boundaries at 20 kb resolution. (D) Box plot showing that the TAD boundary intensity score was lower after *Stella* knockout. (E) Box plot showing the TAD boundary score distribution for the overlapping and specific TAD boundaries.
